# The effect of nursing services management efficiency on nurses’ professional commitment: A cross‐sectional study

**DOI:** 10.1111/inr.13003

**Published:** 2024-06-28

**Authors:** Sevda Arslan Seker, Simge Coskun Palaz

**Affiliations:** ^1^ Nursing Department Munzur University Faculty of Health Sciences Tunceli Turkey; ^2^ Nursing Department Bolu Abant Izzet Baysal University Faculty of Health Sciences Bolu Turkey

**Keywords:** Efficiency, hospital, nurse administrators, nurses, organizational, professional commitment

## Abstract

**Aim:**

To determine the effect of nurses' perceptions of the effectiveness of nursing services management on their professional commitment.

**Background:**

Effective healthcare management is crucial for improving patient care quality. Understanding nurses' perspectives on nursing services management and their commitment provides valuable insights for management strategies, improving well‐being and healthcare outcomes.

**Methods:**

This descriptive, cross‐sectional, and correlational study involved 265 state hospital nurses. Data were collected between November 2020 and June 2021 using descriptive characteristic form, Nursing Services Management Effectiveness Scale, and Nursing Professional Commitment Scale. This study adhered to the STROBE checklist.

**Results:**

Nurses' views on the effectiveness of nursing services management and their professional commitment were found to be influenced by age, professional experience, and institutional positions. The average scores for nursing services management perception and professional commitment were 2.65 ± 0.72 and 66.63 ± 12.40, respectively. A statistically significant positive and low‐level correlation was observed between nurses' nursing services management perception and professional commitment scores. Nurses' positive perception of nursing services management explains 17.1% of the variance in nurses' professional commitment levels.

**Discussion and conclusion:**

This study outlines how nurses' views on effective nursing services management can enhance their professional commitment. Managers should contribute to increase nursing services management efficacy by eliminating deficiencies in management practices to make their institutions successful and to increase their employees’ professional commitment level.

**Implications for nursing and health policy:**

Assuming positive perceptions of nursing management increase nurses' professional commitment, developing policies to boost organizational support is vital. Managers should focus on improving nursing services management to increase organizational success, increase nurses' professional commitment, and achieve the best health outcomes by eliminating management deficiencies.

## INTRODUCTION

Nursing embodies a specialized professional service seamlessly integrated into the intricate tapestry of healthcare, drawing upon a wealth of nursing knowledge and insights. This dynamic role positions nursing at the core of health service organizations, inherently entwined with the principles of management to realize overarching nursing objectives (Kadir et al., 2020; World Health Organization, 2022). Management is defined as the effective and efficient utilization of all managerial resources, involving multiple individuals, to achieve specific objectives through the stages of the management process (Tatar Baykal & Harmancı Seren, 2014). Thus, nursing management is the process of implementing nursing services through nursing staff to provide nursing care, treatment, and security for patients, families, and the community (Kadir et al., 2020).

Nursing services management is integral to achieving organizational objectives by coordinating quality care provision, human resource management, and cost‐effective planning (Almutairi & Bahari, 2022). Effective management has been shown to enhance patient care quality, foster a culture of safety, and reduce hospitalization durations and mortality rates (Khomami & Rustomfram, 2019). Additionally, it promotes professional satisfaction and commitment by cultivating an effective organizational climate and implementing supportive policies (Chang et al., 2019a).

Different definitions of professional commitment describe it as an individual's efforts related to his/her profession, desire to continue the profession, and the power of the individual's identification with the profession (Çetinkaya et al., 2015). Professional commitment, which has significant effects on the quality of care provided in health services, patient care outcomes, and organizational effectiveness, can affect employee turnover intentions (Chang et al., 2019a; Chang et al., 2019b; García‐Moyano et al., 2019), job satisfaction (Chang et al., 2019b; García‐Moyano et al., 2019), performance, absenteeism, and organizational effectiveness (García‐Moyano et al., 2019). It can also improve patient safety (Al‐Hamdan et al., 2018; García‐Moyano et al., 2019) and the quality of care as perceived by patients; furthermore, it has a positive effect on attitudes toward interprofessional cooperation (Galletta et al., 2016).

Ensuring effective nursing services management in healthcare and increasing nurses' commitment to the profession are critical for preventing or solving many problems that nurses face. The relevant literature indicates that nurses experience long and irregular working hours, perceive a decrease in the number of employees, perceive a decrease in organizational justice, have to work for low wages, and heavy workload, can access limited resources in the work environment, and have to live separately from their families in extraordinary situations such as pandemics (Chang et al., 2019a; Labrague & De Los Santos, 2021). Studies have shown that low job satisfaction can lead to low performance and turnover intention; in cases where turnover rates are high, absenteeism or stress can negatively affect business relationships (Acea‐López et al., 2021; Astiti & Surya, 2021).

As part of its responsibilities, nursing services management is required to make any necessary arrangements and take any necessary measures for ensuring the highest levels of job satisfaction among the nursing staff. Ensuring employees’ motivation can increase their job satisfaction and professional commitment. In this context, exploring the relationship between nurses' perceptions of nursing services management effectiveness and their professional commitment becomes essential for advancing both individual well‐being and positive healthcare outcomes. This study sought to outline the perspectives of hospital‐employed nurses on the efficacy of nursing services management and their dedication to the profession. Additionally, it aimed to assess how nurses' perceptions of nursing services management effectiveness influence their professional commitment.

## METHODS

### Study design and participants

A cross‐sectional and correlational study design was utilized. The population of the research consisted of a total of 820 nurses working in four public hospitals in a city center. In the literature, in cases where the universe is known, the sample size is calculated in response to the universe size. Accordingly, it is stated that if the population is 850 people, the sample size will be 265 people (Büyüköztürk et al., 2020). Based on this, the sample in the study was determined as 265 nurses. The inclusion criteria were as follows: working full‐time in an institution for at least six months and volunteering to participate in the study. During this study's implementation process, 320 nurses became participants, but 55 nurses were excluded from the data analysis because they did not complete the questionnaire. The sample size that was suitable for the study was reached. The STROBE checklist was used to ensure that all the relevant aspects of the research process were appropriately reported (Von Elm et al., 2014).

### Data collection

The current researcher collected the relevant data from the participant nurses between November 1, 2020, and June 30, 2021, after obtaining the ethics committee and institutional permission. Due to COVID‐19–related health restrictions, data were collected through an online survey to reduce mobility and maintain social distancing. An anonymous online version of the study questionnaire was created using Google Forms. Data was gatherd online. The researcher met face to face with each staff nurse to explain the purpose of the study, to invite her/him to participate, and to share the data collection link. The first page of the online survey provided an information sheet outlining the study purpose and use of data, and participants were asked to confirm (click a box) if they were willing to proceed. The data collection link was conveyed to the participants through social media, that is, WhatsApp, Facebook, and email. Participation was voluntary. The questionnaires took between 20 and 25 minutes to complete. The completed questionnaires’ information was then transferred into a data set in Excel.

### Instruments

Data were collected using descriptive characteristics form, Nursing Services Management Effectiveness Scale, and Nursing Professional Commitment Scale.

***The descriptive characteristics form*** included four questions for determining the study participants’ sociodemographic characteristics (age, gender, and education level) and the duration of the nurses’ professional experience.

***Nursing Services Management Effectiveness Scale* (NSMES)**: The NSMES, which was developed by Çetinkaya et al. (2019), consists of 38 items and five sub‐dimensions (general management policies, unity of purpose, delegation of authority, hierarchy level, and reporting). Responses to each question are rated on a five‐point Likert scale. The scale evaluation was based on the sub‐dimensions, with a minimum score of 1 and a maximum score of 5. A mean score close to 1 on the sub‐dimensions of the NSMES indicated that the participants' views on nursing services management efficacy were negative, while a mean score close to 5 indicated that their relevant views were positive. The Cronbach's alpha coefficient of the scale was found to be 0.96 in the original study (Çetinkaya Kutun et al., 2019), and it was calculated as 0.94 in the current study, indicating very good reliability.

***Nursing Professional Commitment Scale (NPCS)***: Çetinkaya et al. (2015) conducted a Turkish validity and reliability study to assess the efficacy of this scale (developed by Lu et al., 2000) with regard to nurse participants to determine their professional commitment level (Çetinkaya et al., 2015). The scale included 26 items and three sub‐dimensions (willingness to make an effort, maintaining professional membership, and belief in goals and values). The responses to each question are rated using a four‐point Likert‐type scale. Scores obtained using the scale varied between 26 and 104. The lowest and highest scores that could be obtained from the sub‐dimensions were “willingness to make an effort” (13–52), “maintaining professional membership” (8–32), and “belief in goals and values” (5–20), with higher values indicative of higher levels of professional commitment. The Cronbach's alpha coefficient of the scale was found to be 0.90 in the original study (Çetinkaya et al., 2015), this value was calculated as 0.91 in the current study, indicating very good reliability.

### Data analysis

The IBM SPSS Statistics 23 (Chicago, Illinois, USA) package program was used for data analysis. While evaluating the data, frequency distributions were provided for the categorical variables, and descriptive statistics (mean ± SD) were provided for the numerical variables. In this study, the skewness and kurtosis values of the data (between −1.5 and +1.5) were examined to evaluate whether the study group showed a normal distribution (Fidell et al., 2013). One‐way analysis of variance, Kruskal–Wallis, Mann–Whitney *U*, Pearson correlation coefficient, and simple linear regression analyses methods were used.

### Ethical considerations

The hospital administration provided approval for study implementation (dated 29th September 2020).Relevant ethical approval (2020/04) was obtained from the Bolu Abant İzzet Baysal University Clinical Research Ethics Committee. Permission to use relevant scales in the current study was obtained from the original scale authors via email. Besides the researcher, the online questionnaire forms informed participants about the study purpose, inclusion criteria, confidentiality, voluntariness requirement, and their right to withdraw at any stage of the study. The first part of the form was intended to obtain the nurses’ study participation consent. The confidentiality of the data and the anonymity of the study subjects were maintained. The study was conducted in accordance with the Declaration of Helsinki.

## RESULTS

The participant nurses’ mean age was 31.87 ± 7.55 years; 90.2% of the participants were female, and 9.8% were male. Education‐wise, 62.6% had bachelor's degrees, and 40.8% had 0–5 years of professional experience.

The mean scores of the general management policies, unity of purpose, hierarchy order, and reporting sub‐dimensions of the NSMES showed a statistically significant difference based on the age and professional experience variables (*p* < 0.05). Accordingly, the mean scores of the 18–29 age group were found to be significantly lower than those of the 30–39, 40, and above age groups. The mean scores of the general management policies, unity of purpose, hierarchy order, and reporting sub‐dimensions for nurses with 11–15 years of professional experience and over 21 years of professional experience were significantly higher than the corresponding scores for those with 0–5, 6–10, and 16–20 years of professional experience.

The participants’ mean scores for the hierarchy order sub‐dimension of the NSMES showed a statistically significant difference based on the education level variable (*p* < 0.05). Doctoral graduate nurses’ mean scores for the hierarchy order sub‐dimension were significantly higher than those of bachelor's degree nurses.

The mean total and sub‐dimension scores of the NPCS showed a statistically significant difference based on the age and professional experience variables (*p* < 0.05). Accordingly, the mean scores of nurses in the 30–39 age group were significantly higher than the corresponding scores of those in the 18–29 age group. The mean NPCS scores of nurses with 11–15 years of professional experience were significantly higher than those of nurses with 0–5 and 6–10 years of professional experience.

The mean scores for the willingness to make an effort and sustaining professional membership sub‐dimensions of the NPCS showed a statistically significant difference based on the educational level variable (*p* < 0.05). Accordingly, the mean willingness to make an effort and sustaining professional membership sub‐dimension scores of nurses with at least one doctoral degree were significantly higher than those of nurses with at least one bachelor's degree (Table 1).

**TABLE 1:** Examining the mean scores of Nursing Services Management Efficiency Scale and Nursing Professional Commitment Scale in terms of descriptive characteristics (n= 265)

					NSMES					NPCS			
					General Management Policies	Unity of Purpose	Delegation of Authority	Hierarchy Level	Reporting	Willingness to Make an Effort	Maintaining Professional Membership	Belief in Goals and Values	NPCS Total Score
	Variables		n	%	$\bar{x}$±SD	$\bar{x}$±SD	$\bar{x}$±SD	$\bar{x}$±SD	$\bar{x}$±SD	$\bar{x}$±SD	$\bar{x}$±SD	$\bar{x}$±SD	$\bar{x}$±SD
**Individual characteristics**	**Age** **31.871±7.551 (range 21‐52)**	18‐29^a^	123	46.4	2.18±.76	2.30±.74	3.05±.72	2.31±.74	2.30±.89	30.68±7.15	18.97±4.59	13.86±2.44	63.52±12.46
		30‐39^b^	94	35.5	2.62±.89	2.64±.78	3.18±.77	2.54±.83	2.90±.85	34.22±6.59	20.59±4.46	15.20±2.09	70.02±10.94
		40 and above^c^	48	18.1	2.69±.98	2.86±.88	3.21±.60	2.80±.83	2.91±.82	33.85±7.27	21.81±4.98	14.54±2.16	70.20±12.47
		*Test*			F = 9.965 p = .000	F = 10.513 p = .000	p*>.05*	F = 6.994 p = .001	F = 16.085 p = .000	F = 7.927 p = .000	F = 7.457 p = .001	F = 9.287 p = .000	F = 9.944 p = .000
		*Posthoc(Tukey))*			*a<b‐c* ***(p = 0.000)***					*a<b* ***(p = 0.000)***			
	**Sex**	Female	239	90.2	2.43±.89	2.53±.82	3.12±.72	2.49±.79	2.61±.93	32.72±7.01	20.14±4.71	14.45±2.31	66.18±12.22
		Male	26	9.8	2.35±.76	2.40±.66	3.15±.74	2.41±1.00	2.74±.77	30.57±8.30	19.34±4.93	14.50±2.65	56.10±13.75
		*Test Mann‐Whitney U*			p*>.05*					p*>.05*			U = 2318.000 p = .633
	**Education‐wise**	Vocational School of Health^a^	21	7.9	2.43±.65	2.48±.58	3.16±.56	2.51±.75	2.74±.67	31.33±5.87	19.66±3.49	14.47±1.80	64.50±10.11
		Associate degree^b^	26	9.8	2.46±.92	2.58±.82	3.26±.73	2.53±.82	2.65±1.01	32.53±6.62	18.42±5.64	14.53±2.17	63.91±12.56
		Bachelor's degree^c^	166	62.6	2.29±.77	2.44±.76	3.14±.73	2.55±.78	2.65±89	31.48±6.64	19.98±4.44	14.19±2.50	61.00±10.05
		Post graduate^d^	46	17.4	2.31±.87	2.29±.82	2.98±71	2.14±.86	2.42±.94	35.71±8.65	21.95±5.23	15.15±1.87	69.27±13.75
		Doctoral graduate^e^	6	2.3	2.91±.88	3.16±.89	3.24±1.11	2.66±1.13	2.94±1.58	40.33±3.38	16.16±3.37	16.00±2.36	74.00±12.67
		*Test*			p>.05	p>.05	p>.05	F = 2.464 p = .046	p>.05	F = 5.419 p = .000	F = 3.837 p = .005	F = 2.211 p = .068	F = 2.096 p = .082
		*Posthoc(Tukey/Gabriel)*			***e>c (*p = .046)**					***e>c (*p = .000, *p = 0.005)***			

					NSMES					NPCS			
					General Management Policies	Unity of Purpose	Delegation of Authority	Hierarchy Level	Reporting	Willingness to Make an Effort	Maintaining Professional Membership	Belief in Goals and Values	NPCS Total Score
	Variables		n	%	$\bar{x}$±SD	$\bar{x}$±SD	$\bar{x}$±SD	$\bar{x}$±SD	$\bar{x}$±SD	$\bar{x}$±SD	$\bar{x}$±SD	$\bar{x}$±SD	$\bar{x}$±SD
**Professional characteristics**	**Professional Experience**	0‐5 years^a^	108	40.8	2.19±.79	2.34±.77	3.03±.74	2.29±.75	2.33±.91	31.62±7.22	19.30±4.22	14.02±2.61	64.95±12.39
		6‐10 years^b^	57	21.5	2.39±.77	2.41±.70	3.10±.67	2.52±.73	2.63±.91	31.33±6.92	19.21±5.32	14.47±2.22	65.01±12.47
		11‐15 years^c^	43	16.2	2.79±.98	2.71±.81	3.35±.74	2.65±.91	3.07±.70	35.34±6.51	21.39±4.60	15.37±1.93	72.11±10.73
		16‐20 years^d^	21	7.9	2.68±.99	2.80±1.03	3.24±.82	2.56±.96	2.79±1.04	31.47±7.74	19.19±4.66	14.28±1.87	64.95±11.42
		21 years and above^e^	36	13.6	2.61±.94	2.83±.79	3.13±.63	2.73±.80	2.87±.82	34.27±6.88	22.61±4.37	14.75±2.12	71.63±11.93
		*Test*			F = 4.894 p = .001	F = 4.200 p = .003	p>.05	F = 2.886 p = .023	F = 16.085 p = .000	F = 3.263 p = .013	F = 5.080 p = .001	F = 2.708 p = .027	F = 3.298 p = .012
		*Posthoc(Benforroni)*			**c‐e >a‐b‐d (p = *0.03)***					***c>a‐b (p = 0.012)***			

p<.05

The nurses’ mean scores were 2.65 ± 0.72 for NSMES and 66.63 ± 12.40 for NPCS. When the sub‐dimensions of the total NSMES were examined, it was found that the nurses scored 2.44 ± 0.89 in the general management policies sub‐dimension, 2.53 ± 0.81 in the unity of purpose sub‐dimension, 3.14 ± 0.71 in the delegation of authority sub‐dimension, 2.49 ± 0.81 in the hierarchy order sub‐dimension, and 2.65 ± 0.91 in the reporting sub‐dimension. When the nurses’ scores for the NPCS sub‐dimensions were examined, it was found that they obtained 32.33 ± 7.18 for the willingness to make an effort sub‐dimension, 19.89 ± 4.84 for the maintaining professional membership sub‐dimension, and 14.40 ± 2.35 for the belief in goals and values sub‐dimension (Appendix 1).

A statistically significant positive and low‐level correlation was found between the nurses’ total scores in the NSMES and the NPCS (*r* = 0.413**, *p* < 0.01). There was a statistically significant, positive, and low‐level relationship between the scores for general management policies (*r* = 0.404**, *p* < 0.01), Unity of Purpose (*r* = 0.421**, *p* < 0.01), and reporting (*r* = 0.419**, *p* < 0.01), which are the NSMES sub‐dimensions, and the NPCS scores. There was a statistically significant, positive, and low‐level relationship between the delegation of authority (*r* = 0.292**, *p* < 0.01) and hierarchy order (*r* = 0.252**, *p* < 0.01) sub‐dimensions of the NSMES and the NPCS scores (Table 2).

**TABLE 2:** The relationship between nurses' views on the nursing services management efficiency and level of professional commitment.

		NPCS total score
**NSMES total score**	*r*	0.413^a^
General management policies	*r*	0.404^a^
Unity of purpose	*r*	0.421^a^
Delegation of authority	*r*	0.292^a^
Hierarchy level	*r*	0.252^a^
Reporting	*r*	0.419^a^

^a^ Correlation is significant at the 0.01 level (2‐tailed).

It was found that nurses' positive perception of nursing services management had a positive and weakly significant effect on nurses’ professional commitment levels. The analysis results showed that 17.1% of the total variance in nurses’ professional commitment level could be explained by nurses' positive perception of nursing services management, which is the independent variable in the model (*R* = 0.413; *R* ^2^ = 0.171; *p* < 0.05) (Table 3).

**TABLE 3:** Regression analysis of nursing services management efficiency to predict nurses' levels of professional commitment.

Independent variable	Dependent variable	*B*	SH	β	*t*	*P*
NSMES total score	NPCS total score	47,911	2.884	0.413	16.615	0.00
*F* = 45.252, *R* = 0.413, *R* ^2 ^= 0.171, *p* = 0.00						

## DISCUSSION

This study examined the effect of nurses' perceptions of the effectiveness of nursing services management on their professional commitment.

### Nurses' perceptions of the effectiveness of nursing services management

The research findings revealed that nurses perceive the effectiveness of nursing services management at a moderate level, with the highest scores attributed to the delegation of authority sub‐dimension. The study conducted by Çetinkaya Kutun et al. (2019) supported the current study's findings, particularly highlighting the conscious approach of nurses toward their workplace managerial organizational structures, shared responsibilities in nursing services management, and active delegation of authority. However, when the other sub‐dimensions were examined, certain inadequacies were observed. The relevant literature underscores the significance of managerial processes, such as positive verbal expressions by nursing managers toward the profession (Çelik & Yıldız, 2018), constructive relational styles and attitudes (Er & Altuntaş, 2014), sharing institutional management policies and objectives (Çetinkaya Kutun et al., 2019), and supporting independent decision‐making (Khomami & Rustomfram, 2019), in enhancing management effectiveness, motivating employees, and fostering effective work environments. Research findings underscore the imperative of refining managerial processes within nursing services.

### Nurses' demographic factors and NSMES sub‐dimensions

This study found that there was no difference between scores for gender and the mean scores for the NSMES sub‐dimensions; however, a significant difference was observed between educational level and hierarchy order; age and professional experience and general management policies; unity of purpose, hierarchy order, and reporting sub‐dimensions. Specifically, nurses holding doctoral degrees exhibited significantly higher scores in the hierarchy order sub‐dimension compared with those with bachelor's degrees. Çetinkaya Kutun et al. (2019) found a statistically significant difference between education level and the general management policies, delegation of authority, and reporting sub‐dimensions. Professionalization in the profession increases with increasing education levels. Education levels can increase professionalization in the profession. It is expected that professional members with a higher education level and advanced knowledge will strive to represent nursing services management strongly in the institution. Professional members with these characteristics can use the desired skills such as leadership, strategic decision‐making, professional commitment, continuous development, and evidence‐based practices in nursing services management. Moreover, they are also more likely to be actively involved in representing and advancing the profession within the institution. Furthermore, the study findings showed a significant difference between age and professional experience and the NSMES sub‐dimensions of general management policies, unity of purpose, hierarchy order, and reporting. Çetinkaya Kutun et al. (2019) found a statistically significant difference between age and the reporting sub‐dimension. This difference could be attributable to the following factors of young nurses: their knowledge is up to date; their professional awareness is higher; they may be assigned based on their education level, skills, and areas of expertise; and they can adapt to the policies, goals, and objectives determined by the institution more easily. The same study determined that the difference in the averages of the NSMES sub‐dimensions based on the duration of professional experience was statistically significant; furthermore, a difference was observed between nurses with professional experience of 11–15 years and those with professional experience of 16 years or more (Çetinkaya Kutun et al., 2019). Factors such as the gradual development of communication skills with an increase in working time, familiarity with the institution and its functioning, and professional experience can contribute to this difference. Furthermore, nurses' perceived stress related to teamwork, workload perception, and managerial attitudes could change with increasing age. Over time, as nurses gain experience and adapt to working life, they may consequently try to eliminate factors related to stress sources.

### Nurses' professional commitment and implications

In this study, nurses' professional commitment was moderate. Previous studies’ findings have been (Al‐Hamdan et al., 2018; Can, 2021; Dönmez & Karakuş, 2019; Pekeren & Başdaş, 2022) similar to the findings of this study. Unlike this study, other studies have found that nurses’ professional commitment is above average (Duran et al., 2021; Tuna & Sahin, 2021) and high (Parnikh et al., 2022; Tarhan et al., 2022). Occupational commitment is expressed as an important factor that determines people's working behaviors, including the extent of employees’ professional commitment and their thoughts about their professions. Avaialble evidence suggests that employees’ increased professional commitment is associated with many professional, managerial, and organizational outcomes, such as increased job performance and job satisfaction, decreased turnover intention, and strengthened interdisciplinary communication (Duran et al., 2021; Tuna & Sahin, 2021). This suggests that the actions of nurse managers with regard to making nursing services visible within the organization, directing employees in line with the goals and objectives of the organization, and creating a suitable working environment and working conditions can be effective in increasing nurses’ professional commitment. Employees with high professional commitment can provide services that reflect a holistic care approach by prioritizing patients without neglecting their responsibilities toward themselves and their profession, increasing employees’ satisfaction, and effectively developing employees’ creativity (García‐Moyano et al., 2019). The findings of the present study indicate that nurses' professional commitment falls below the desired level, and they encounter challenges and difficulties that impact both their working environment and, potentially, the effectiveness of managerial practices.

### Nurses' demographic factors and NPCS sub‐dimensions

In this study, no significant difference was found between gender, level of education, and the mean total NPCS scores, while there was a significant difference in age and professional experience. Similar to this study's findings, another study did not find any significant difference between gender and total scores on professional commitment (Dönmez & Karakuş, 2019). Unlike this study, some previous studies found that there was a significant difference between gender and the total NPCS scores; furthermore, female nurses had higher professional commitment than male nurses (Al‐Hamdan et al., 2018; Parnikh et al., 2022; Pekeren & Başdaş, 2022; Tuna & Sahin, 2021). Moreover, a study by Tuna and Sahin (2021) showed that there was a significant difference between the NCPS sub‐dimensions of willingness to make an effort and sustaining professional membership among female nurses. Professional commitment in nursing is characterized by a nurse's belief in and acceptance of the values of the profession. It involves a dedicated effort to realize these values, a willingness to enhance one's professional capabilities, and is determined to persist in the nursing profession. However, in this study, no significant difference emerged when considering the comparison with the total NPCS score. This featured result prompts further exploration into the relationship between educational levels and specific aspects of professional commitment, as opposed to its overall measure. Future research could investigate thoroughly into understanding the factors contributing to this distinction, offering valuable insights into the complexities of the relationship between education levels and professional commitment.

Different studies have indicated that education level and professional commitment are related (Duran et al., 2021; Mersin et al., 2020), and others have indicated that they are not related (Al‐Hamdan et al., 2018; Mersin et al., 2020; Parnikh et al., 2022). This current study observed a difference between the level of education and the willingness to make an effort and maintaining professional membership NPCS sub‐dimensions. Previous studies have stated that high professional competence and education levels can increase nurses’ professional commitment (Duran et al., 2021; Numminen et al., 2016). Mersin et al. (2020) found that, as the level of education increased, the mean scores for nurses' beliefs about goals and values also increased. A study by Tuna and Sahin (2021) showed a significant difference between the level of education and the NPCS sub‐dimensions, but no significant difference was observed in comparison with the total score. Another study found that, as nurses’ education level increased, their self‐confidence, awareness, analytical thinking, and communication skills improved (Rahman et al., 2015). These skills are renowned to have a positive impact on patient care outcomes. In light of these data, the current study's findings align with the anticipated correlation between higher levels of education and the development of important skills, suggesting a potential positive impact on patient care outcomes.

This current study found that the professional commitment of nurses in the 30–39 age group was statistically significant compared with that of nurses in the 18–29 age group. Previous studies have indicated that age and professional commitment are related (Numminen et al., 2016), and others have indicated that they are not related (Al‐Hamdan et al., 2018; Dönmez & Karakuş, 2019; Mersin et al., 2020; Parnikh et al., 2022). The current study results indicated that professional experience is related to professional commitment; this finding is similar to those of this current study (Numminen et al., 2016; Tuna & Sahin, 2021). In contrast, Al‐Hamdan et al. (2018) reported that total length of service as a nurse was not associated with professional commitment. Our research showed that nurses’ level of professional commitment is higher, especially in the 30–39 age group; furthermore, the professional commitment of nurses in the 11–15 years’ experience group was significantly higher than that of nurses with 0–5 and 6–10 years of professional experience. This suggests an improvement in the professional commitment of nurses who have gained a certain amount of experience in their professional field and have developed the ability to cope with difficulties related to human relations and their working environment.

### Relationship between nurses' perceptions of the effectiveness of nursing services management and professional commitment

A statistically significant, positive, and low‐level relationship was observed between the total NSMES scores of the nurses and the total NPCS scores. The literature has emphasized that a number of factors, such as feeling valuable, taking part in the decision‐making process, job satisfaction, being motivated and supported by managers, having limited access to information, equipment, and adequate resources, having a positive working environment, participating in effective corporate management policies, teamwork, strengthening communication and cooperation, receiving continuous learning opportunities, professional status, adequate human workforce, and wage policies, are associated with professional commitment (Al‐Hamdan et al., 2018; Chang et al., 2019a; Dönmez & Karakuş, 2019; García‐Moyano et al., 2019). Almutairi and Bahari (2022) have stated that managerial competence is related to organizational commitment. Nurses’ professional commitment affects patient safety, patient‐centered care, and quality of care (Al‐Hamdan et al., 2018; Almutairi & Bahari, 2022; Parnikh et al., 2022).

The current study results showed that nurses' positive perception of nursing services management had a positive and weakly significant effect on nurses' professional commitment. Professional commitment is crucial, influencing various aspects such as job satisfaction, organizational effectiveness, and turnover intention (Chang et al., 2019a; García‐Moyano et al., 2019). Previous studies have demonstrated its significant impact on turnover intention (Chang et al., 2019a), with a notable contribution of approximately 34% toward job satisfaction in nursing (Barac et al., 2018). Nurse's positive perceptions of management are believed to enhance professional commitment, potentially leading to greater employee retention and organizational loyalty.

### Limitations of the study

The current study results were limited to the scales used in the data collection phase and were based on the nurses' self‐reports. Survey studies may be insufficient for revealing important information because they can cause Type II errors. Appropriate sampling methods may have limited the representativeness of the population. The results of this cross‐sectional study were limited to reflecting the conditions experienced during the data collection process. The current study data were collected using an online survey method, and participation from nurses who could not use this technology well may have been insufficient. The study results may have been affected by factors such as the working environments of the hospitals where the sample was selected and the attitude of the hospital management.

## CONCLUSIONS

Nurse perceptions of nursing services management effectiveness and professional commitment were moderate and correlated with factors such as age, education, and experience. Positive perceptions of management were weakly linked to higher professional commitment levels. The study underscores the importance of further research on managerial effectiveness in nursing, emphasizing its potential to improve patient care, safety, and employee satisfaction. Recommendations include increasing training opportunities, involving nurses in decision‐making processes, and fostering supportive work environments. Future research should focus on assessing management effectiveness, developing strategies to enhance it, and exploring the role of education in this context.

### Implications for nursing and health policy

The study's findings carry significant policy implications for nursing and health policy:

*Improving nursing services management*: This study underlines the need for interventions to improve nursing services management in healthcare organizations. Policy initiatives should focus on improving work environments and promoting effective managerial practices that contribute to better outcomes for both nurses and patients. Also, it is considered important to develop and implement policies that increase organizational support levels.
*Professional Commitment*: Health policies can emphasize strategies to increase nurses' commitment to their profession, such as targeted educational programs and supportive work environments.
*Education and Training*: Given the link between educational level and professional commitment, policies should advocate for continuous education and training opportunities for nurses, including updates in the nursing education curriculum to provide managerial skills and competence.

## AUTHOR CONTRIBUTIONS

*Study design*: Sevda Arslan Seker and Simge Coskun Palaz. *Data collection*: Simge Coskun Palaz. Data analysis: Sevda Arslan Seker and Simge Coskun Palaz. *Study supervision*: Sevda Arslan Seker. *Manuscript writing*: Sevda Arslan Seker and Simge Coskun Palaz. *Critical revisions for important intellectual content*: Sevda Arslan Seker and Simge Coskun Palaz.

## CONFLICT OF INTEREST STATEMENT

The authors declared no potential conflicts of interest with respect to the research, authorship, and/or publication of this article.

## FUNDING INFORMATION

This research received no specific grant from any funding agency in the public, commercial, or not‐for‐profit sectors.

## ETHICS STATEMENT

Permission to use relevant scales in the current study was obtained from the original scale authors via email (cetinkaya2015@yahoo.com.tr, feyza.cetinkaya@gmail.com). Ethical approval (2020/04) was obtained from the Bolu Abant Izzet Baysal Unıversıty Clinical Research Ethics Committee.

## Supporting information

Supporting Information
