# Supplementary material for: The effect of nursing services management efficiency on nurses’ professional commitment: A cross‐sectional study
Source: Int Nurs Rev. 2024 Jun 28;72(1):e13003. doi: 10.1111/inr.13003 (PMC11740411; doi:10.1111/inr.13003)
Supplement: Supplementary file 1 — Supporting Information [file INR-72-0-s001.docx]

**APENDIX 1. Mean Scores of Nurses' Nursing Services Management Efficiency Scale and Sub-Dimensions of the Nursing Professional Commitment Scale (n= 265)**

|  | **Items** | **±SD** | **Min-Maks** | **Cronbach's α** |
| --- | --- | --- | --- | --- |
| **NSMES Total** | 38 | 2.655±0.727 | 1-4.30 | 0.942 |
| General Management Policies | 10 | 2.442±0.891 | 1-4.40 | 0.924 |
| Unity of Purpose | 11 | 2.532±0.815 | 1-4.82 | 0.910 |
| Delegation of Authority | 9 | 3.146±0.711 | 1-4.78 | 0.830 |
| Hierarchy Level | 5 | 2.499±0.819 | 1-4.40 | 0.738 |
| Reporting | 3 | 2.654±0.916 | 1-4.33 | 0.714 |
| **NPCS Total** | 26 | 66.630±12.409 | 31-96 | 0.910 |
| Willingness to Make an Effort | 13 | 32.333±7.187 | 13-52 | 0.879 |
| Maintaining Professional Membership | 8 | 19.891±4.842 | 8-32 | 0.819 |
| Belief in Goals and Values | 5 | 14.405±2.354 | 5-20 | 0.600 |
